# Lifetime cost-effectiveness analysis osseointegrated transfemoral versus socket prosthesis using Markov modelling

**DOI:** 10.1302/2633-1462.53.BJO-2023-0089.R1

**Published:** 2024-03-15

**Authors:** Jeffrey D. Voigt, Benjamin K. Potter, Jason Souza, Jonathan Forsberg, Danielle Melton, Joseph R. Hsu, Benjamin Wilke

**Affiliations:** 1 Walter Reed National Military Medical Center, Bethesda, Maryland, USA; 2 Uniformed Services University of the Health Sciences, Bethseda, Maryland, USA; 3 Ohio State University Wexner Medical Center, Columbus, Ohio, USA; 4 Johns Hopkins University, Baltimore, Maryland, USA; 5 Sibley Memorial Hospital, Washington DC, USA; 6 University Colorado School of Medicine, Aurora, Colorado, USA; 7 Atrium Health, Charlotte, North Carolina, USA; 8 Mayo Clinic, Jacksonville, Florida, USA

**Keywords:** Cost-effectiveness, Markov modelling, Transfemoral amputation, Prosthesis, OPRA, socket prostheses, Transfemoral Amputation, osseointegrated prosthesis, sensitivity analysis, arthroplasty, Short-Form Health Survey, prosthetic, mechanical complications, comorbidities, EQ-5D

## Abstract

**Aims:**

Prior cost-effectiveness analyses on osseointegrated prosthesis for transfemoral unilateral amputees have analyzed outcomes in non-USA countries using generic quality of life instruments, which may not be appropriate when evaluating disease-specific quality of life. These prior analyses have also focused only on patients who had failed a socket-based prosthesis. The aim of the current study is to use a disease-specific quality of life instrument, which can more accurately reflect a patient’s quality of life with this condition in order to evaluate cost-effectiveness, examining both treatment-naïve and socket refractory patients.

**Methods:**

Lifetime Markov models were developed evaluating active healthy middle-aged male amputees. Costs of the prostheses, associated complications, use/non-use, and annual costs of arthroplasty parts and service for both a socket and osseointegrated (OPRA) prosthesis were included. Effectiveness was evaluated using the questionnaire for persons with a transfemoral amputation (Q-TFA) until death. All costs and Q-TFA were discounted at 3% annually. Sensitivity analyses on those cost variables which affected a change in treatment (OPRA to socket, or socket to OPRA) were evaluated to determine threshold values. Incremental cost-effectiveness ratios (ICERs) were calculated.

**Results:**

For treatment-naïve patients, the lifetime ICER for OPRA was $279/quality-adjusted life-year (QALY). For treatment-refractory patients the ICER was $273/QALY. In sensitivity analysis, the variable thresholds that would affect a change in the course of treatment based on cost (from socket to OPRA), included the following for the treatment-naïve group: yearly replacement components for socket > $8,511; cost yearly replacement parts OPRA < $1,758; and for treatment-refractory group: yearly replacement component for socket of > $12,467.

**Conclusion:**

The use of the OPRA prosthesis in physically active transfemoral amputees should be considered as a cost-effective alternative in both treatment-naïve and treatment-refractory socket prosthesis patients. Disease-specific quality of life assessments such as Q-TFA are more sensitive when evaluating cost-effectiveness.

Cite this article: *Bone Jt Open* 2024;5(3):218–226.

## Introduction

The osseointegrated prosthesis for rehabilitation of amputees (OPRA, Integrum, Sweden) was FDA-approved in December 2020 under a pre-market approval (PMA) designation. The OPRA is indicated for use in transfemoral amputees (TFAs) due to trauma or cancer, or in those who are anticipated to have problems with a conventional socket prothesis. These indications include patients with recurrent skin infections or ulcerations, pain with socket use, restricted mobility, volume fluctuations in the stump, and socket retention problems due to excessive perspiration or a short residual limb.^1-3^

The OPRA is an implant system employing skeletal anchoring of the prosthesis along with a skin-penetrating device, and is typically implanted in a two-stage surgical process. Over the past four to five years, there have been several comparison studies that have examined costs only,^4-6^ quality of life (QoL),^7-10^ and the cost-effectiveness^11,12^ of an osseointegrated prosthesis compared to a traditional socket-suspended prosthesis. These analyses, however, have either been short-term (i.e. ≤ 20 years),^6,8^ incomplete in capturing costs,^4^ non-USA-specific,^6-9,11,12^ and non-disease-/condition-specific as it relates to the QoL.^11,12^ Additionally, prior cost-effectiveness assessments of OPRA versus a traditional socket have been undertaken only in amputees who were treatment-refractory to a socket prosthesis.^11^ Longer-term five- to 15-year outcome data have recently been published, which provide additional insights into the longevity, mechanical complications, and patient-reported outcomes using OPRA implants.^13,14^

The purpose of this cost-effectiveness analysis was to evaluate an OPRA versus a socket prosthesis in treatment-naïve (i.e. those amputees who have not had a prosthesis; the primary outcome) and treatment-refractory (those who have a problem with a socket prosthesis and switch to an OPRA; the secondary outcome) healthy, physically active, middle-aged unilateral TFA males in the USA.

## Methods

A digital literature search was performed on 1 April 2022 to retrieve studies published from 2001 to the present, on both socket and OPRA-type transfemoral prostheses using search criteria focused on clinical, economic, and cost outcomes. The Supplementary Material shows the search criteria used. The variables and distributions identified in relevant studies were used in the modelling process.

Markov health state transition models (TreeAge Pro 2022) were built to represent two treatment options - comparing the OPRA and socket prosthesis for treatment-naïve and treatment-refractory patients. In treatment-naïve patients, amputees who were not able or unlikely to tolerate a socket were treated with the OPRA prosthesis. Health states in each model included: steady state (OPRA and socket); failure/intolerable (socket); implant surgery and recovery (OPRA); living with an uncomfortable prosthesis (socket); abandons prosthesis (socket), treatment of mechanical complications (OPRA and socket); and death. For treatment-refractory patients, all health states were the same except the patient was assumed to have already been treated with the socket prosthesis, transitioning from socket to OPRA as part of the treatment paradigm.

Patients were assumed to be physically active, middle-aged (aged 46 years) healthy males with a unilateral TFA. The model was run for 20, 30, and up to 42 years (based on insurance life expectancy).^15^ Direct costs for medical care were evaluated. For these costs, Medicare reimbursements for procedures and services related to implantation, complications, and follow-up were used. The model assumed manufacturer pricing for the prostheses, and associated costs for mechanical complications and replacement parts (Supplementary Tables i and ii).

For QoL assessments, the disease-specific instrument for persons with a transfemoral amputation (Q-TFA) global score^16^ associated with each health state was used from the literature.^7,9,13,14,16,17^ Each Q-TFA was aggregated over time into a single number that could be compared across different types of treatments representing the length and QoL, termed quality-adjusted life-years (QALYs).

Probabilities for health states and complications were derived from published studies representative of the type of patient who was entered into the Markov Model. Costs and QoL were discounted at 3% annually.^18^ Discounting reflects a decrease/loss in value that occurs over time when there is a delay in realizing a benefit (i.e. effectiveness outcome) or when costs are incurred over time.

**Fig. 1:**
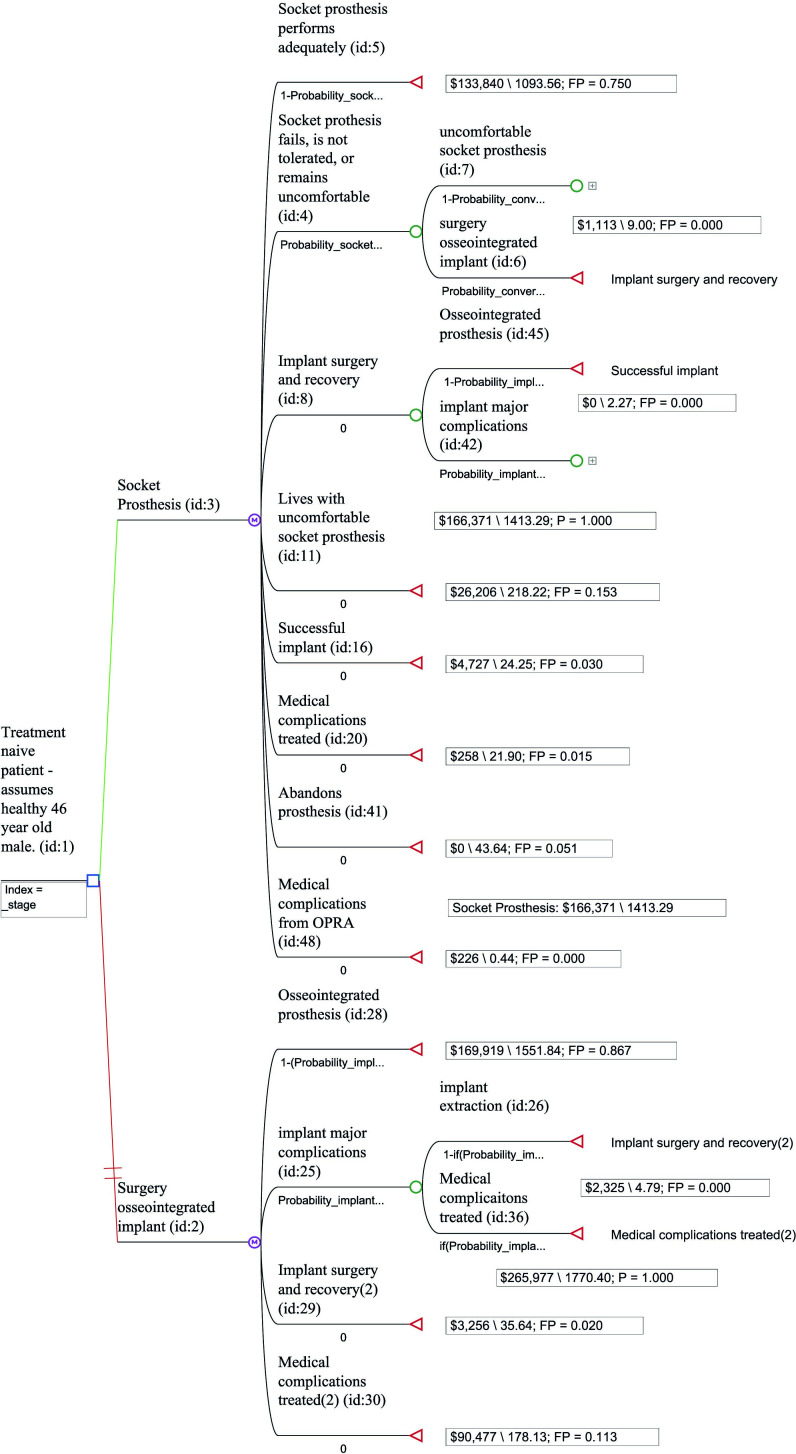
Treatment-naïve model structure.

**Fig. 2:**
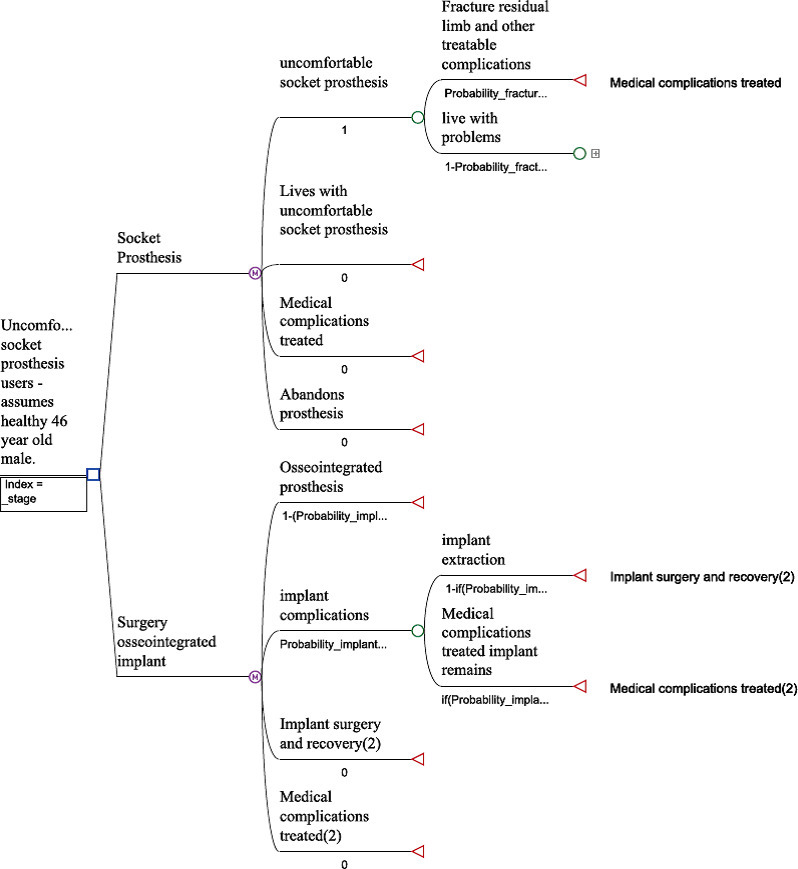
Treatment-refractory model structure.

**Fig. 3:**
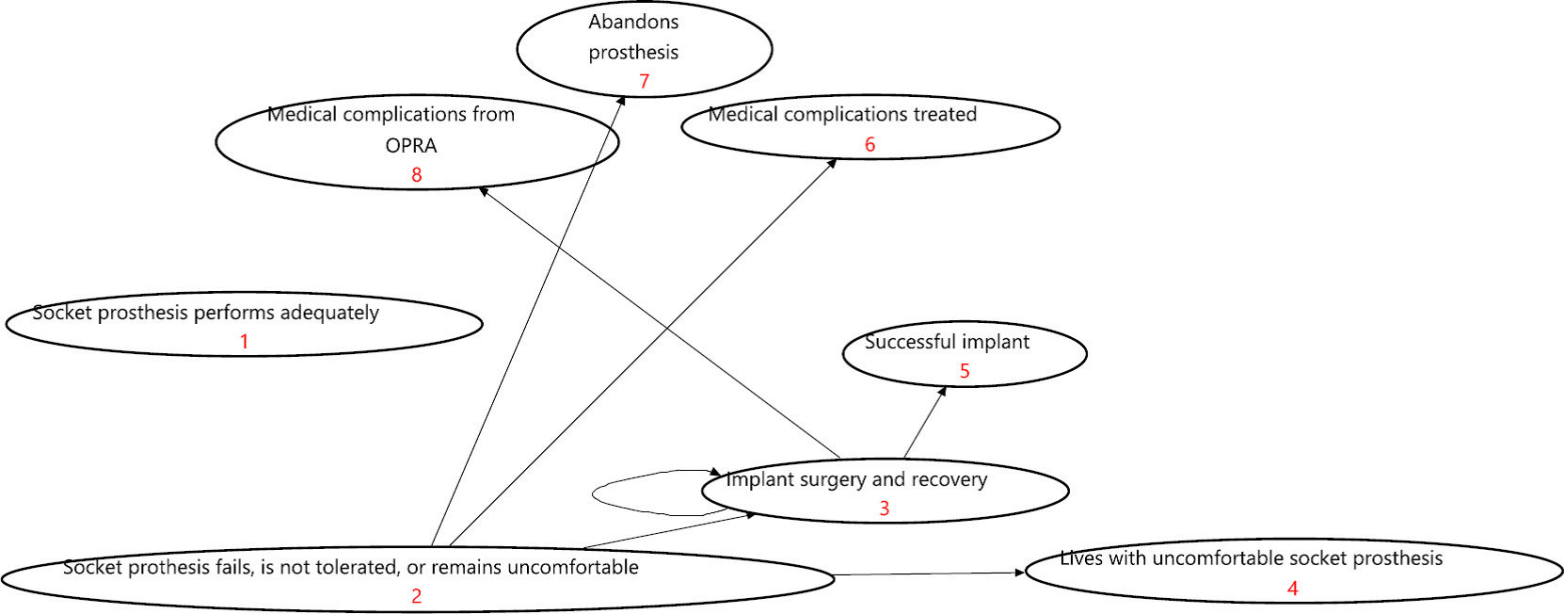
Markov model health state transition diagram. OPRA, osseointegrated prosthesis for rehabilitation of amputees.

**Fig. 4:**
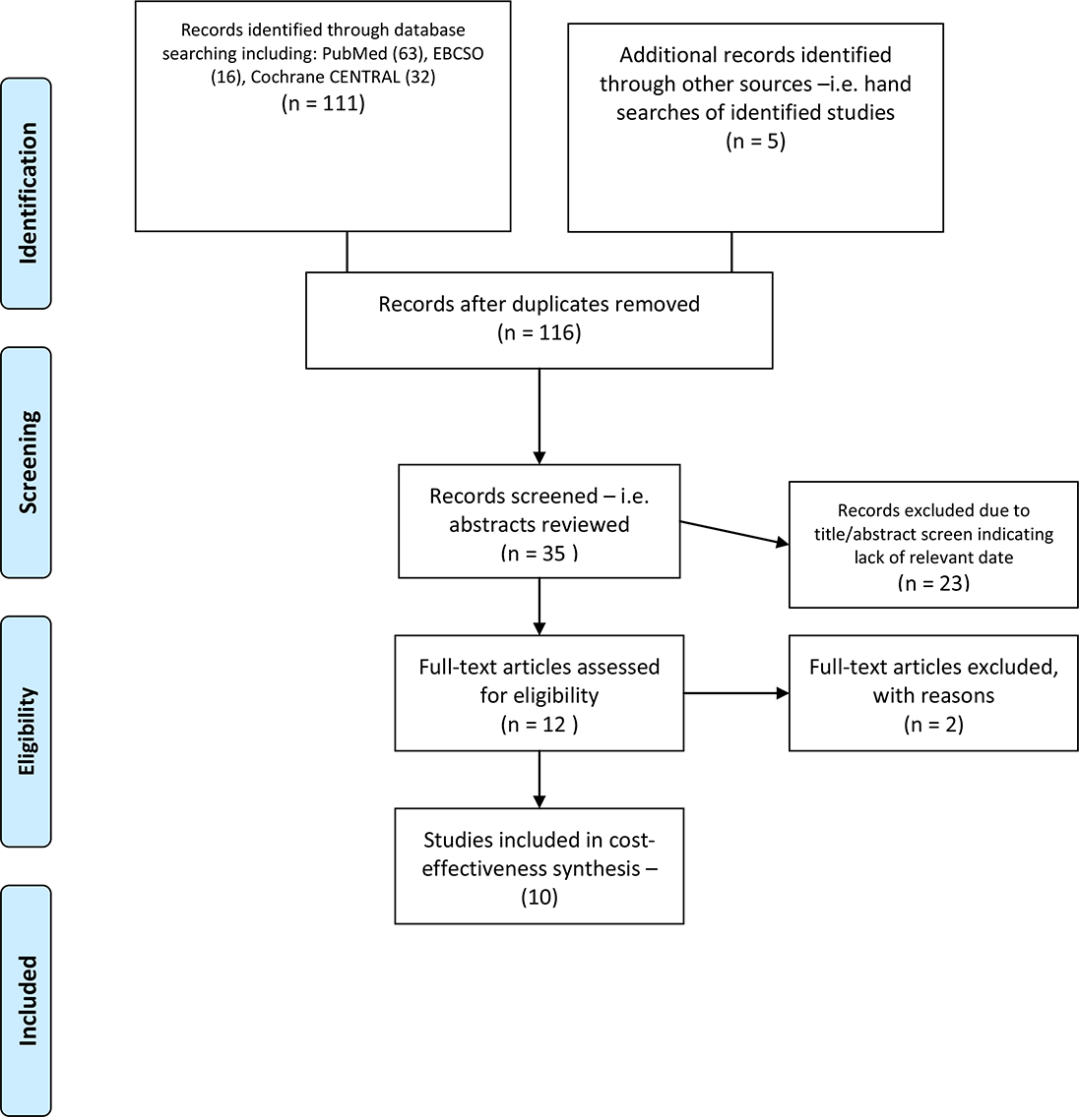
Preferred Reporting Items for Systematic Reviews and Meta-Analyses diagram of studies used in Markov model.

Figure 1 (treatment-naïve) and Figure 2 (treatment-refractory) show each of the model’s structures. Supplementary Tables iii to vi show variables and distributions used in each model. As an example of how a patient would transition from one health state to the next, Figure 3 shows a Markov model health state transition diagram for a treatment-naïve socket prosthesis patient. Meanwhile, Figure 4 shows the Preferred Reporting Items for Systematic Reviews and Meta-Analyses (PRISMA) diagram of studies used in the Markov models. A summary of these papers can be found in Supplementary Table vii.

### Statistical analysis

Base case analyses estimated the QALYs and costs for each intervention to identify additional costs per QALY gained (incremental cost-effectiveness ratio; ICER). Analysis of uncertainty was undertaken via: one-way sensitivity (with literature supported variation) in order to determine which variables had an effect in changing a type of treatment based on costs; and probabilistic sensitivity (to capture uncertainty in all parameters simultaneously) using Monte Carlo sampling (10,000 times).

## Results

Base case estimates for both treatment-naïve and treatment-refractory models are shown in Table I (for 20, 30, and 42 years). The specific costs associated with the initial OPRA implant and socket prosthesis are found in Supplementary Tables i and ii.

**Table I.** Incremental cost-effectiveness analysis, in US$.

Disease state	Timeframe (years)	Costs	QALYs	ICER
Treatment-naïve socket	20	$105,753	901	
Treatment-naïve OPRA	20	$176,910	1,125	$333
Treatment-naïve socket	30	$138,199	1,175	
Treatment-naïve OPRA	30	$224,583	1,470	$293
Treatment-naïve socket	42	$166,371	1,413	
Treatment-naïve OPRA	42	$265,977	1,770	$279
Treatment-refractory socket	20	$77,533	785	
Treatment-refractory OPRA	20	$176,910	1,125	$292
Treatment-refractory socket	30	$102,146	1,033	
Treatment-refractory OPRA	30	$224,583	1,470	$280
Treatment-refractory socket	42	$123,518	1,250	
Treatment-refractory OPRA	42	$265,977	1,770	$273

ICER, incremental cost-effectiveness ratio; OPRA, osseointegrated prosthesis for rehabilitation of amputees; QALY, quality-adjusted life-year; QALYs, quality-adjusted life-years.

Table II shows the value of those variables in which the alternative type of treatment would be chosen upon their cost variation (for 42-year lifespan).

**Table II.** One-way sensitivity analysis.

Variable	Model	Value at which OPRA would be the preferred course of treatment based on cost savings	Costs used in Markov model
Yearly cost replacement parts and service socket prosthesis (Figure 5)	Treatment-naïve	> $8,511	$3,850
Yearly cost replacement parts and service OPRA (Figure 6)	Treatment-naïve	< $1,758	$6,606 ± 1,764
Yearly cost replacement parts and service socket (Figure 7)	Treatment-refractory	> $12,467	$3,850

OPRA, osseointegrated prosthesis for rehabilitation of amputees.

**Fig. 5:**
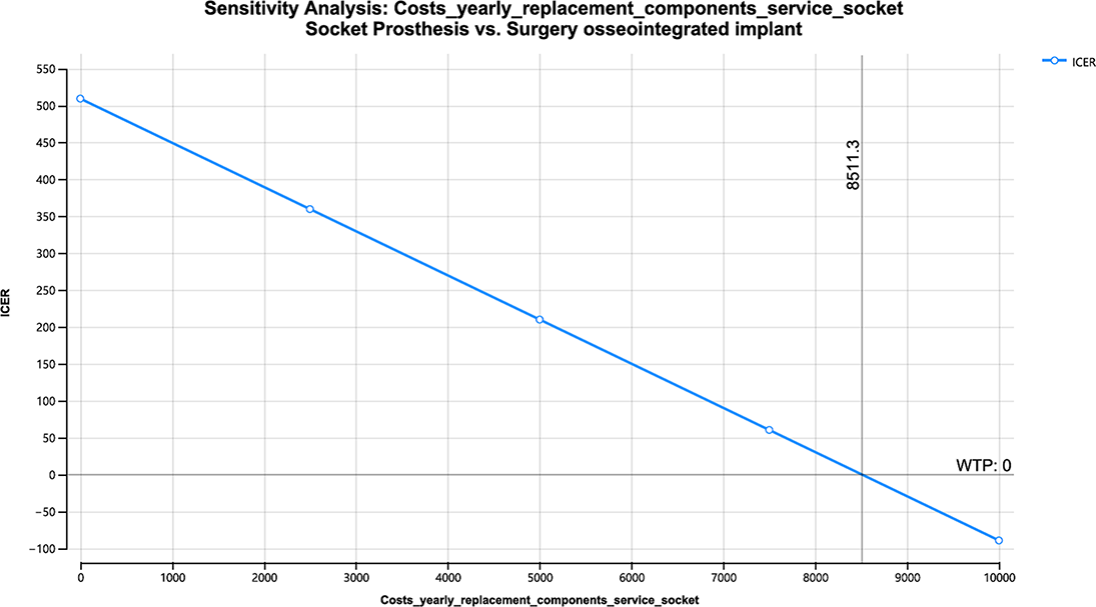
One-way sensitivity analysis yearly cost replacement parts and service socket – treatment-naïve. ICER, incremental cost-effectiveness ratio; WTP, willingness to pay.

**Fig. 6:**
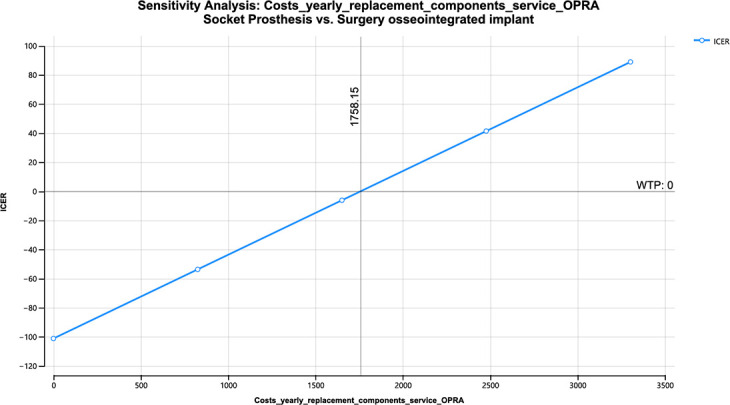
One-way sensitivity yearly costs replacement parts and service osseointegrated prosthesis for rehabilitation of amputees (OPRA) – treatment-naïve. ICER, incremental cost-effectiveness ratio; WTP, willingness to pay.

**Fig. 7:**
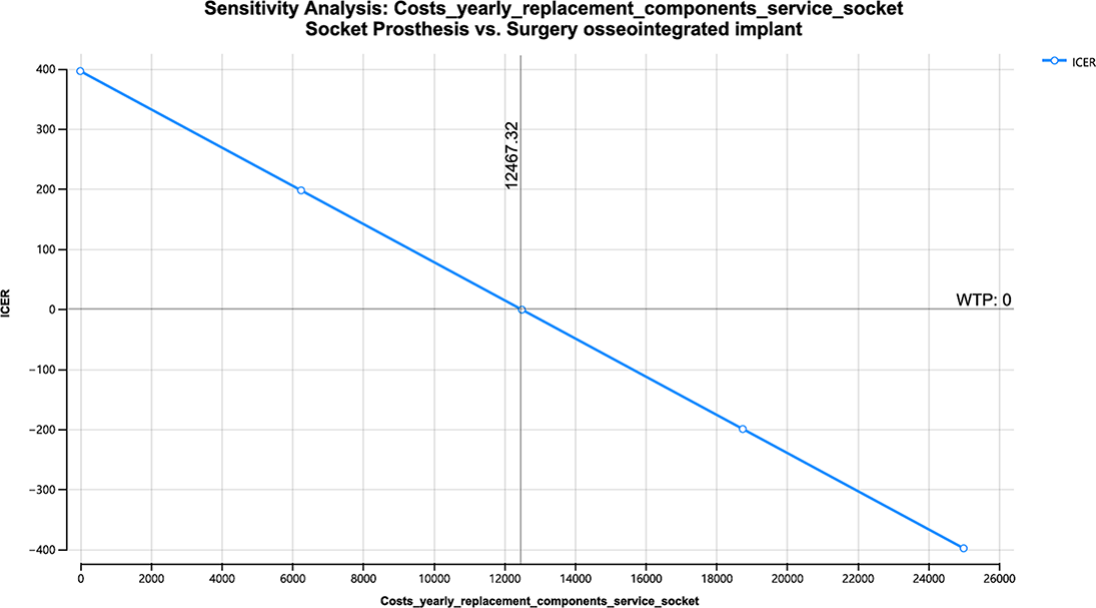
One-way sensitivity yearly costs replacement parts and service socket – treatment-refractory. ICER, incremental cost-effectiveness ratio; WTP, willingness to pay.

**Fig. 8:**
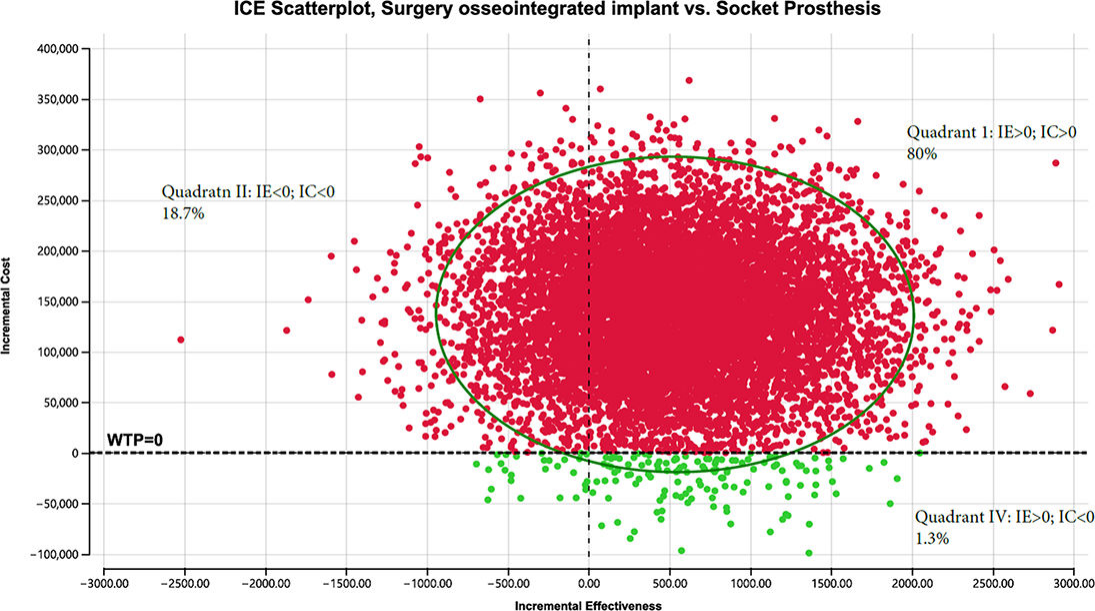
Incremental cost-effectiveness (ICE) scatter plot, treatment refractory. IC, incremental costs; IE, incremental effectiveness; WTP, willingness to pay.

**Fig. 9:**
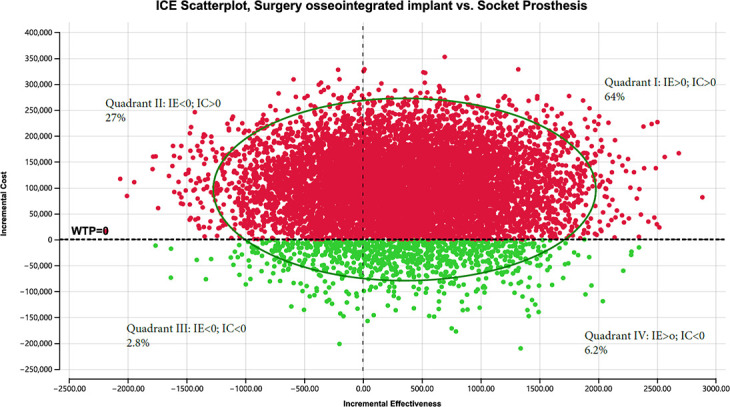
Incremental cost-effectiveness (ICE) scatter plot, treatment-naïve. IC, incremental costs; IE, incremental effectiveness; WTP, willingness to pay.

Probabilistic sensitivity revealed that in the majority of cases (80% for treatment-refractory (Figure 8) and 63.7% for treatment-naïve (Figure 9)), OPRA resulted in improved effectiveness and increased cost versus the socket prosthesis, with ICERs of $279/QALYs for treatment-naïve and $273/QALYs for treatment-refractory patients.

## Discussion

Based on the ICER findings in both treatment-naïve and treatment-refractory patients, the ICER values of $273 to $279/QALY in using the OPRA prosthesis in the USA are within an accepted ICER threshold value of < $50,000/QALY.^19,20^

The OPRA system permits direct skeletal control of a prosthetic limb. It is currently the only device of its kind to date that has obtained FDA clearance. It has previously been used largely at Walter Reed National Military Medical Centre for service members who have sustained injuries,^21^ and is now offered at other USA medical centres. OPRA is most often used as a salvage therapy to improve function after exhausting conventional strategies. Interestingly, the finding that OPRA is most cost-effective when used in the treatment-naïve setting suggests that repetitive failure of socket prostheses should likely not be a prerequisite by third party payers for OPRA’s use.

Recent publications have added more informed outcomes and costs to the present analysis versus prior analyses.^11^ These include additional data captured prospectively over five and 15 years on mechanical complications/costs with OPRA and Q-TFA longer-term assessments.^13,14^ These studies found statistically significant improvements over baseline on global QoL (as measured by Q-TFA) and mechanical failures due to increased use of the OPRA prosthesis. These data were added to the present analysis and provided additional insights that prior cost-effectiveness studies do not.

Differences exist in the current analysis versus prior ones. First, prior analyses used a period of 20 years.^11^ Second, other analyses have used more generic QoL assessments (36-Item Short-Form Health Survey questionnaire (SF-36)) and EuroQol five-dimension three-level questionnaire (EQ-5D-3L),^11,12,22^ which have not been found to be as sensitive in capturing condition-specific differences.^11^ As stated by the National Institute for Health and Care Excellence,^23^ generic health instruments such as the EQ-5D may not be the most appropriate due to a lack of sensitivity to condition-specific maladies. In making a case for a generic health instrument being inappropriate, evidence should be provided that key dimensions of health are missing (lack of content validity). Additionally, evidence of an instrument’s poor performance on construct validity (lack of responsiveness to the instrument) should also be shown.

The EQ-5D and SF-36 do not measure the amount of prosthetic use, comfort of the prosthesis and improved function, especially over time (i.e. a lack of content validity).^24^ Furthermore, general population health instruments do not measure the amputee’s ability to use, and satisfaction with, the prosthesis – the most important aspects to an amputee.^25^ A 2011 systematic review of the literature on QoL in amputees stated that amputee-specific validated instruments on the QoL are needed, which capture the unique facets of daily living that amputees encounter. This would make QoL assessments more relevant, comprehensive, and useful to those making decisions on the use of prostheses.^26^

The Q-TFA is a validated health instrument for evaluating non-elderly healthy amputees using a prosthesis.^10^ The Q-TFA also addresses the issues of function/mobility (which is part of the overall global score). Due to its lack of sensitivity, the EQ-5D demonstrates no statistically significant difference in global health scores when comparing OPRA to socket, while the Q-TFA does.^27^ Further, the Short-Form six-dimension questionnaire (SF-6D, derived from the SF-36 for use in cost-effectiveness analysis) provides a single “general health index”, and has been noted not to be sensitive in capturing detailed differences with specific groups such as transfemoral amputees.^11,28^ For the above reasons, Q-TFA was chosen as the effectiveness outcome in the analysis. Table III shows assessments made with Q-TFA, EQ-5D, and SF-36 over time in the patient populations, and their statistical differences on QoL for osseointegrated and socket prosthesis.

**Table III.** Questionnaire for Persons with a Transfemoral Amputation, EuroQol five-dimension questionnaire, and 36-Item Short-Form Health Survey questionnaire assessment over time.

Study	Study type	Comparisons	Findings
Hagberg et al, 2008^29^	Two-year follow-up on osseointegrated prosthesis	Q-TFA and SF-36 assessments at baseline and 2 years	Q-TFA global score significantly different at 2 years from baseline (p = 0.002). SF-36 general health score no different at 2 years from baseline (p = ns)
Brånemark, et al, 2014^17^	Follow-up on osseointegreated prosthesis for TFA at baseline and 2 years	Q-TFA and SF-36 assessments at baseline and 2 years	Q-TFA global score significantly different at 2 years from baseline (p < 0.001). SF-36 general health score no different at 2 years from baseline (p = ns)
Hagberg et al, 2014^8^	Two-year follow-up on osseointegrated prosthesis	Q-TFA and SF-36 assessments at baseline, and 1 and 2 years	Q-TFA global score significantly different from baseline at 1 and 2 years (p < 0.001). SF-36 global significantly different, albeit less sensitive than Q-TFA (p = 0.007). This SF-36 difference represented a minimal important difference which is the smallest difference considered of relevance to a patient.
Brånemark et al, 2019^13^	Follow-up on osseointegreated prosthesis for TFA at baseline, and 2 and 5 years	Q-TFA and SF-36 assessments at baseline, and 2 and 5 years	Q-TFA global score significantly different at 2 and 5 years from baseline (p < 0.0001). SF-36 general health score no different at 2 and 5 years from baseline (p = ns)
Pospeich et al, 2020^7^	Matched cohort (osseointegrated vs socket prosthesis) for TFA	Q-TFA and EQ-5D-3L assessments in osseointegrated vs socket prothesis amputees	Statistically signficant differences found between osseointegrated and socket prosthesis on Q-TFA global (p = 0.02); no statistical difference in EQ-5D-3L (p = 0.479)

EQ-5D-3L, EuroQol five-dimension three-level questionnaire; ns, not specified; OPRA, osseointegrated prosthesis for rehabilitation of amputees; Q-TFA, Questionnaire for Persons with a Transfemoral Amputation.

Another difference versus prior analyses is that this analysis includes a QoL assessment for those amputees who abandoned their prosthesis (in the treatment-naïve group) on the assumption that they were not converted to the OPRA prosthesis. This lowered the QoL for socket prosthetic users relative to OPRA.^11^

The cost of healthcare in the USA differs considerably compared to other countries. In fact, USA healthcare costs are 50%^12^ to 100%^30,31^ higher here than in previously published cost-effectiveness analyses.^11,12^ These factored into the present ICER analysis and resulted in higher overall costs, but were countered by higher QALYs using the Q-TFA. The use of Q-TFA is most relevant to this population, i.e. non-elderly physically active patients, and focuses on mobility and prosthetic use.^8^ Thus, the cost per QALY findings in the present study were significantly less than in prior analyses,^11^ and well within costs/QALY considered to be of high value.^32^

Strengths of this analysis include being the first analysis to evaluate USA cost-effectiveness of TFA prostheses. It is also the first analysis to use a disease- or condition-specific QoL measure (Q-TFA). In addition, data analysis was used on a population of patients who would benefit from an OPRA prosthesis: healthy, active, middle-aged males. This analysis also included a health state of non-use of a prostheses due to intolerance (in the treatment-naïve analysis), which others did not. The non-socket prosthetic user health state should be included as it is a distinct possibility for a transfemoral amputee.^10^

These findings should also be evaluated in the context of the study’s limitations. Although it sought to identify costs and outcomes in the medical literature related to healthy high-intensity users (e.g. with minimal to no comorbidities), data used in this analysis (albeit small) may have included patients with comorbidities such as diabetes or vascular diseases, thereby confounding the Q-TFA assessments, complications, and costs. Another limitation is that societal (indirect) costs were not included in the analysis. Other analyses have stated that the use of an OPRA prosthesis would be even more cost-effective than a socket type due to the ability of a patient to return to work sooner, and work more efficiently, based on a higher level of function.^11^ Moreover, the model reflects usage in healthy active males. We would, however, expect similar costs per QALY in healthy active females. It was assumed that the representative patient entered into the Markov model had a normal life expectancy (42 additional years of life) based on statements from prior studies.^14,33,34^ However, this may not be the case. Thus, 20 and 30 years of additional life spans were evaluated. Lastly, it was assumed that there was no state of non-use in the OPRA prosthesis as in the socket prosthesis. This assumption was made due a lack of identified literature on this issue.

As in other analyses, the costs of an OPRA implant are higher than with a socket prosthesis. However, the Q-TFA global score was also considerably higher versus generic health instruments, which is consistent with the data found in the literature.^8,13,17^ In summary, the ICER of OPRA was found to be highly cost-effective for healthy, active, middle-aged TFA males.

**Take home message**

- For active transfemoral amputees, the OPRA prosthesis should be the prosthesis of choice.

- The OPRA prosthesis provides improved quality of life in these individuals at a mimimal increased cost compared to a socket prosthesis.

## Data Availability

The data that support the findings for this study are available to other researchers from the corresponding author upon reasonable request.
